# The Mre11 Nuclease Is Critical for the Sensitivity of Cells to Chk1 Inhibition

**DOI:** 10.1371/journal.pone.0044021

**Published:** 2012-08-24

**Authors:** Ruth Thompson, Ryan Montano, Alan Eastman

**Affiliations:** 1 Department of Pharmacology and Toxicology, The Geisel School of Medicine at Dartmouth, Hanover, New Hampshire, United States of America; 2 Norris Cotton Cancer Center, Lebanon, New Hampshire, United States of America; University Medical Center Hamburg-Eppendorf, Germany

## Abstract

The Chk1 kinase is required for the arrest of cell cycle progression when DNA is damaged, and for stabilizing stalled replication forks. As a consequence, many Chk1 inhibitors have been developed and tested for their potential to enhance DNA damage-induced tumor cell killing. However, inhibition of Chk1 alone, without any additional exogenous agent, can be cytotoxic. Understanding the underlying mechanisms of this sensitivity is critical for defining which patients might respond best to therapy with Chk1 inhibitors. We have investigated the mechanism of sensitivity in U2OS osteosarcoma cells. Upon incubation with the Chk1 inhibitor MK-8776, single-stranded DNA regions (ssDNA) and double-strand breaks (DSB) begin to appear within 6 h. These DSB have been attributed to the structure-specific DNA endonuclease, Mus81. The Mre11/Rad50/Nbs1 complex is known to be responsible for the resection of DSB to ssDNA. However, we show that inhibition of the Mre11 nuclease activity leads, not only to a decrease in the amount of ssDNA following Chk1 inhibition, but also inhibits the formation of DSB, suggesting that DSB are a consequence of ssDNA formation. These findings were corroborated by the discovery that Mre11-deficient ATLD1 cells are highly resistant to MK-8776 and form neither ssDNA nor DSB following treatment. However, once complimented with exogenous Mre11, the cells accumulate both ssDNA and DSB when incubated with MK-8776. Our findings suggest that Mre11 provides the link between aberrant activation of Cdc25A/Cdk2 and Mus81. The results highlight a novel role for Mre11 in the production of DSB and may help define which tumors are more sensitive to MK-8776 alone or in combination with DNA damaging agents.

## Introduction

High fidelity DNA replication is essential for the maintenance of genomic stability and cell survival. Cells have therefore evolved intricate checkpoint pathways to ensure the repair of any DNA lesions prior to progression through the cell cycle. Checkpoint kinase 1 (Chk1) is a vital mediator of the S and G2 checkpoints and it is well characterized as being essential for cell survival in the response to many DNA damaging agents [1]–[4]. However, more recent studies have revealed a role for Chk1 in normal S phase progression [5]. Chk1 inhibition in unperturbed human cells can result in the stabilization of Cdc25A and the activation of cyclin dependent kinases (CDKs) [6]. This increased CDK activity causes increased replication origin firing, and DNA-damage accumulates in S-phase most likely due to the aberrant upregulation of replication initiation [7]. Despite the increased origin firing in Chk1-deficient cells, replication fork progression is dramatically reduced [8], [9] and consequently, it has been suggested that Chk1 promotes replication fork progression in normal S phase through the control of replication origin firing [10].

Inhibition of Chk1 has been shown to induce regions of single-stranded DNA (ssDNA), RPA binding to ssDNA and the formation of double strand breaks (DSB) in normal S phase [7]. Replication fork collapse has been proposed as the reason behind S phase-specific DNA damage, and the DNA endonuclease Mus81 has recently been demonstrated as the source of DSB following Chk1 inhibition [11]. However, the DNA substrate for Mus81 cleavage is unknown and this observation does not account for the appearance of regions of ssDNA. The Mre11/Rad50/Nbs1 (MRN) complex functions as a DNA damage sensor and is responsible for the recruitment of ATM to the sites of DSB [12]. The MRN complex also promotes the processing of DSB to ssDNA [13]. We therefore, hypothesized that the Mre11 nuclease could play a role in the production of ssDNA following Chk1 inhibition.

Here we show that the Chk1 inhibitor MK-8776 (previously known as SCH900776) induces phosphorylation of RPA and H2AX in U2OS cells. The Mre11 inhibitor mirin suppresses both these effects. Moreover, the Mre11-deficient cell line ATLD1 was inherently resistant to Chk1 inhibition but could be sensitized through ectopic expression of Mre11. These findings suggest a novel role for Mre11 in the production of DNA DSB following Chk1 inhibition.

## Results

### Chk1 inhibition results in rapid accumulation of ssDNA and DSB in U2OS cells

Since discovering the checkpoint inhibitory activity of UCN-01 over 15 years ago [14], we have performed extensive experiments on the activation of Chk1 by DNA damaging agents and its inhibition by UCN-01, and more recently by MK-8776 [15]. These observations led to the realization that some cell lines are highly sensitive to the inhibition of Chk1 as a single agent.

**Figure 1:**
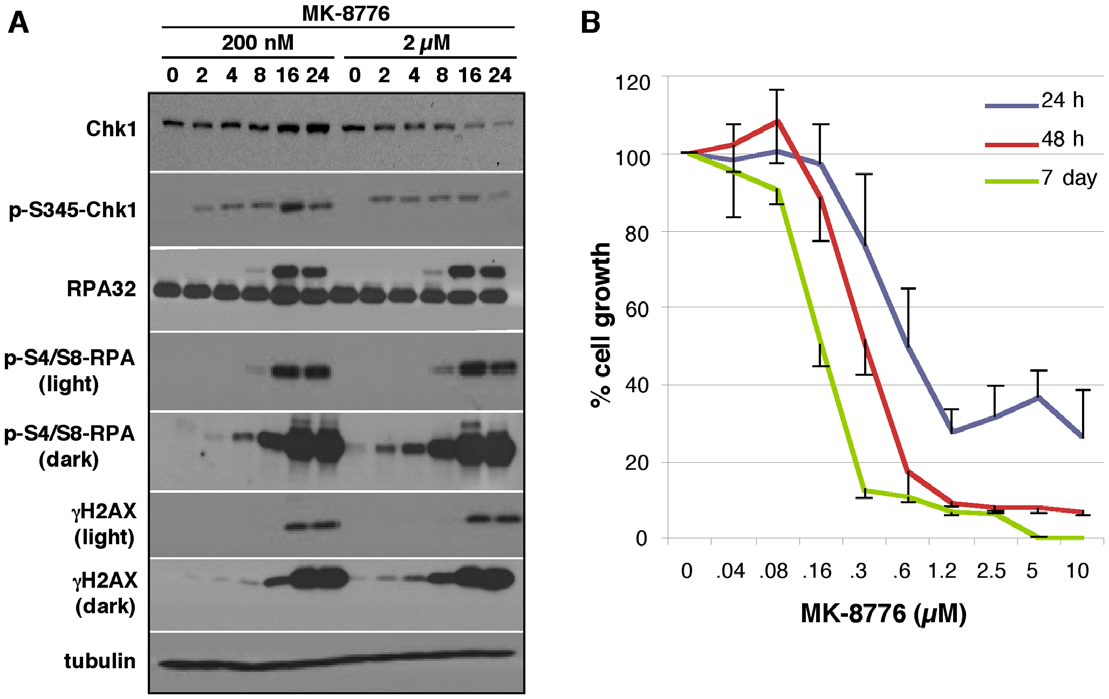
MK-8776 induces DNA damage and S phase arrest in U2OS cells. A. Cells were incubated with 200 nM or 2 µM MK-8776 for 0–24 h then analyzed by western blotting for markers of DNA damage. B. Cells were incubated with MK-8776 for 24 h or 48 h then allowed to recover in drug-free media, or incubated continuously for 7 days with MK-8776. Total DNA content per well was then assessed as a measure of cell growth. Error bars (shown only in one direction for clarity) represent the standard error of 3 independent experiments.

To investigate the role of Chk1 in unperturbed cell cycle progression we incubated U2OS cells with two concentrations of MK-8776, selected based on our previous findings that 2 µM MK-8776 enhances the cytotoxic effects of hydroxyurea in most cell lines but 200 nM was sufficient in more sensitive cell lines such as U2OS [15]. Western blotting revealed that MK-8776 induced phosphorylation of Chk1 at serine 345 at both concentrations as early as 2 h after administration. It has been suggested that this phosphorylation is due to the loss of Chk1-mediated feedback inhibition of ATR [16]. Phosphorylation of H2AX (indicative of DSB formation; see below) and accumulation of RPA phosphorylation at the S4/S8 site began to appear at 4 h and was dramatically elevated by 16 h (Fig. 1A). The phosphorylation of RPA is also observed as a band with retarded electrophoretic mobility in blots of total RPA.

**Figure 2:**
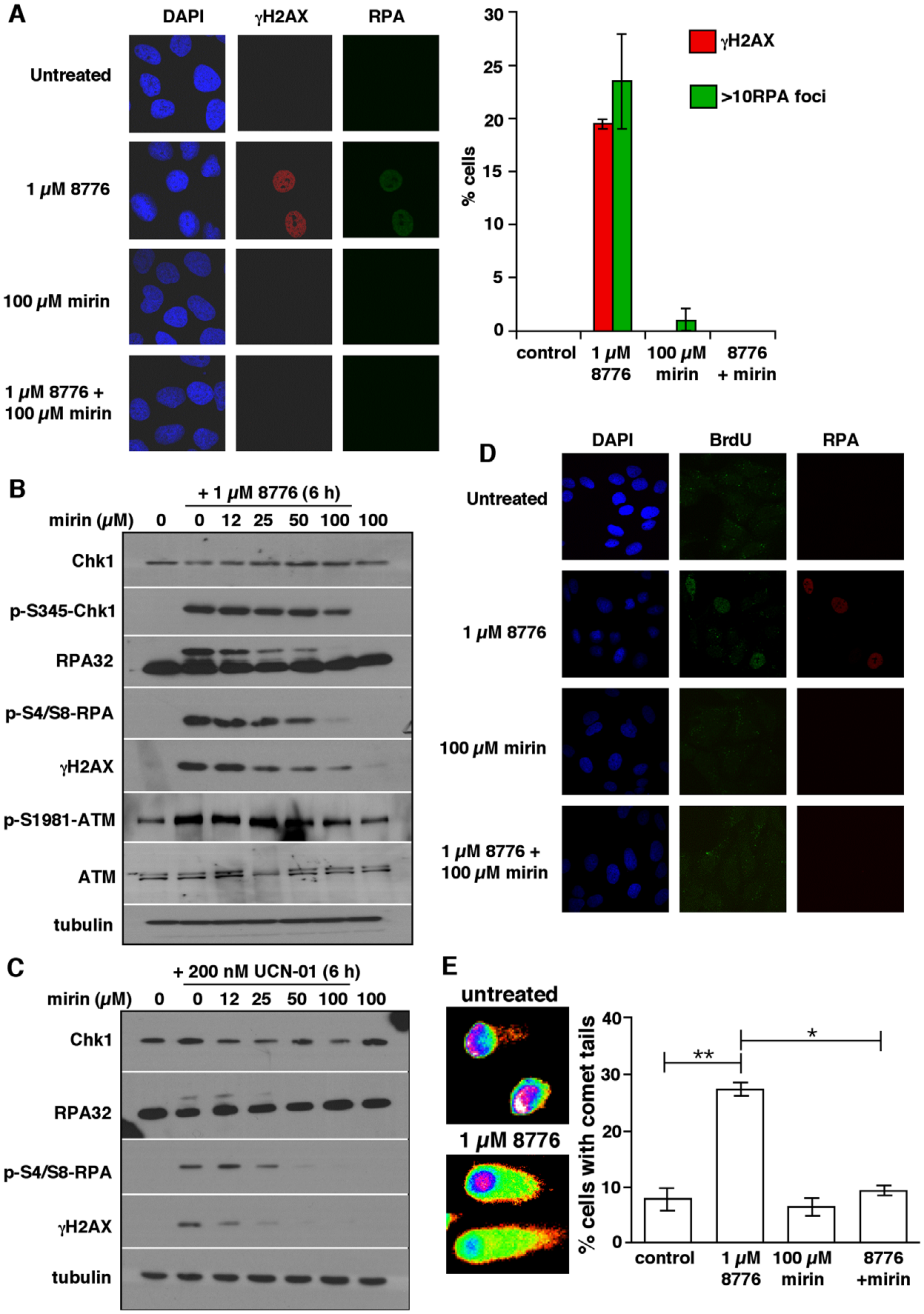
The DNA damage induced by MK-8776 is dependent on Mre11. A. U2OS cells grown on coverslips were incubated with 1 µM MK-8776, 100 µM mirin or both for 6 h then analyzed for γH2AX and RPA foci by confocal microscopy. 100 cells were scored for each condition and are presented as histograms on the right. Bars represent the standard error of 3 independent experiments. B. U2OS cells were co-incubated with MK-8776 and mirin for 6 h and analyzed by western blotting. C. U2OS cells were co-incubated with UCN-01 and mirin for 6 h and analyzed by western blotting. D. Cells were prelabeled with BrdU, then incubated for 6 h with 1 µM MK-8776, 100 µM mirin or both. At harvest, cells were stained with antibodies against BrdU (without DNA denaturation) and RPA. E. U2OS cells were incubated for 6 h with 1 µM MK-8776, 100 µM mirin or both then analyzed using the neutral comet assay. Examples of negative and positive comet tails are shown. Histograms represent cells with DSB based on increased tail moment. Error bars represent the SEM of 2 independent experiments. Significance analyzed using the paired t-test; * = p<0.05, **  = p<0.01.

Analysis by confocal microscopy revealed dramatic γH2AX pan-nuclear staining in response to just 6 h of Chk1 inhibition (Fig. 2A, second row), a phenomenon which has been previously documented following the inhibition or depletion of Chk1 [7]. Furthermore, the cells showing pan-nuclear γH2AX staining (approximately 20%) were, for the most part, positive for RPA foci (>10 foci/cell). The fact that ssDNA and DSB were occurring in the same cells led us to hypothesize that one may be a precursor for the other. There were a few cells positive for RPA but not γH2AX suggesting that RPA foci may appear first.

Our previous work has shown that, compared to a panel of ten cell lines, U2OS cells are very sensitive to Chk1 inhibition by MK-8776 as a single agent [15]. Here, U2OS cells were incubated with MK-8776 for 24 or 48 h, then allowed to grow for an additional 5–6 days (Fig. 1B). Alternatively, cells were incubated continuously for 7 days. The growth curves are fairly similar in all cases demonstrating that the maximum growth suppression is elicited within the first 24 h, and the cells are unable to recover thereafter.

It has been shown that the γH2AX induced by Chk1 inhibition occurs exclusively in cells actively replicating their DNA [17]. We confirmed this was the case by pulse-labeling cells with EdU to stain S phase cells and then incubating with MK-8776 for 6 hours. Cells were then stained for γH2AX and EdU and analyzed by confocal microscopy (data not shown). Collectively, these results suggest that inhibition of Chk1 causes DNA damage in cells actively replicating their DNA.

### The Mre11 inhibitor mirin prevents MK-8776-induced DNA damage

Chk1 inhibition induces DSB which have been attributed to the Mus81 endonuclease [11]. Whether the production of ssDNA regions is a cause or consequence of DSB remains unknown. It is well documented that the MRN complex is recruited to DSB and is required for the processing of DSB to ssDNA to which RPA binds [13], [18]–[20]. To determine whether the ssDNA formed following Chk1 inhibition is Mre11 dependent, we co-incubated cells with MK-8776 and the Mre11 inhibitor mirin. Immunofluorescence showed that the γH2AX pan-nuclear staining and the RPA foci induced by 1 µM MK-8776 were completely inhibited by co-treatment with 100 µM mirin (Fig. 2A). Western blotting revealed that, while there was little change in the amount of phospho-Chk1 at serine-345, the higher concentrations of mirin reduced phospho-RPA, phospho-ATM and γH2AX induced by MK-8776 (Fig. 2B). These concentrations are consistent with those previously shown to inhibit the Mre11 nuclease in cells [21].

To determine whether the damage previously shown to be induced by other Chk1 inhibitors such as UCN-01 is also inhibited by mirin, we repeated the experiment shown in Fig. 2B using 200 nM UCN-01 instead of 1 µM MK-8776 (Fig. 2C). As observed with MK-8776, UCN-01 also induced phosphorylation of RPA and H2AX and both were inhibited by mirin, demonstrating their Mre11 dependence.

To confirm that the induction of RPA phosphorylation and appearance of RPA foci were due to the appearance of regions of ssDNA, we incubated cells with BrdU then used an anti-BrdU antibody to detect incorporated BrdU in the absence of DNA denaturation, such that only endogenous ssDNA is detected [7]. There was 100% concordance between cells with RPA foci and BrdU staining (Fig. 2D, Row 2), demonstrating that RPA foci are indicative of regions of ssDNA. No BrdU staining was seen when cells were co-treated with MK-8776 and mirin (Fig. 2C, Row 4) further supporting our conclusion that Mre11 is required for the production of ssDNA following Chk1 inhibition. The neutral comet assay was also performed to confirm the induction of DSB (Fig. 2E). These data corroborated the γH2AX immunofluorescence showing that approximately 25% of the cells had comet tails following treatment with MK-8776 and mirin reduced the number of cells with tails to that of the untreated control. Taken together, these results suggest that the formation of ssDNA following Chk1 inhibition is Mre11-dependent and occurs upstream of DSB.

**Figure 3:**
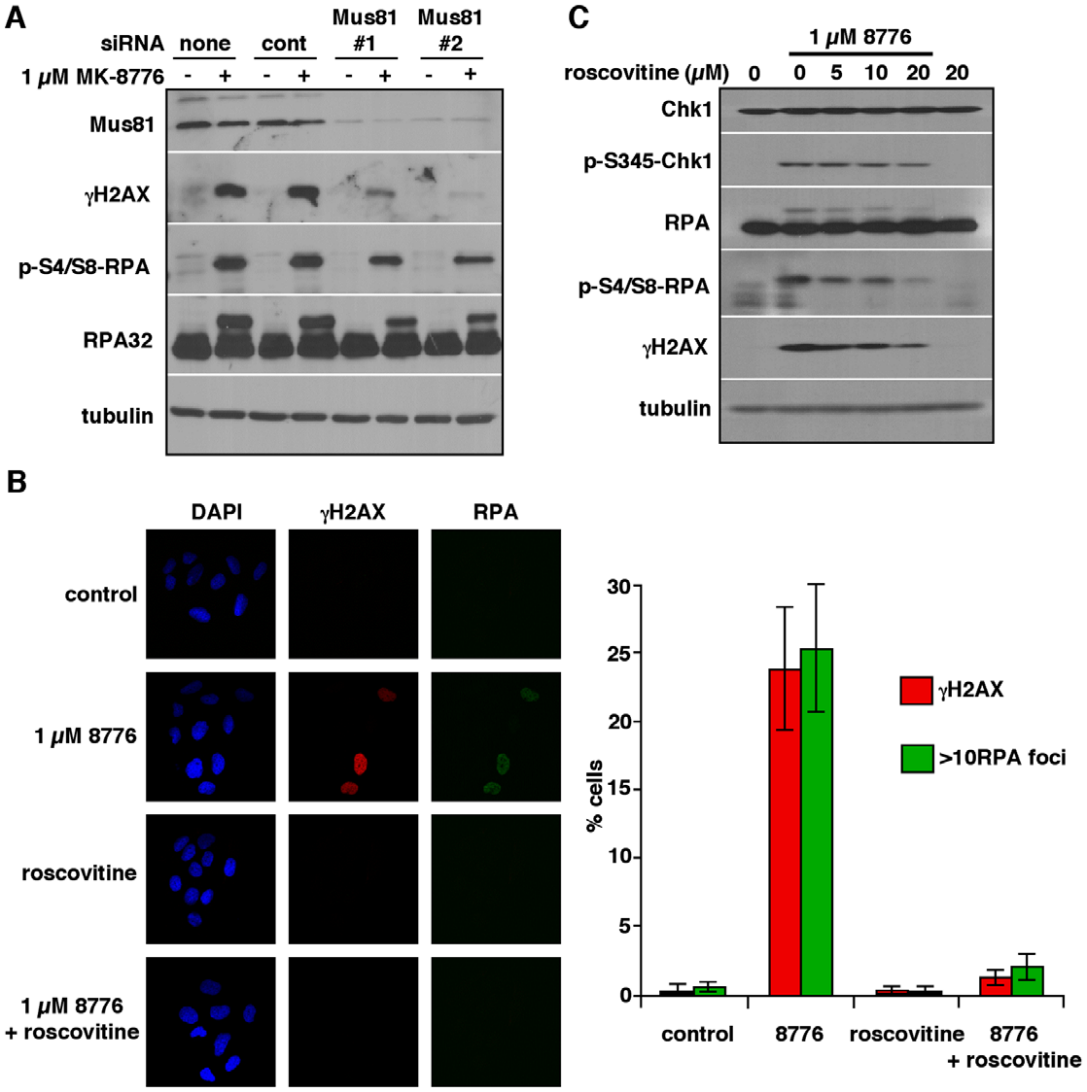
The role of Mus81 and CDK2 in MK-8776-induced DNA damage. A. Cells were transfected with siRNA against Mus81 then analyzed by western blotting for markers of DNA damage following a 6-h treatment with MK-8776. B. U2OS cells grown on coverslips were incubated with 1 µM MK-8776, 20 µM roscovitine or both for 6 h then stained with the indicated antibodies and analyzed by confocal microscopy. 100 cells were scored for each condition and are presented as histograms. Error bars represent the standard error of 2 independent experiments. C. U2OS cells were co-incubated with MK-8776 and roscovitine for 6 h and analyzed by western blotting.

DSB following Chk1 inhibition have been shown to be dependent on the structure specific DNA endonuclease Mus81 [11]. To confirm that ssDNA was occurring upstream and independently of DSB we performed siRNA knockdown of Mus81 in U2OS cells prior to 6 h incubation with MK-8776 (Fig. 3A). Depletion of Mus81 dramatically reduced γH2AX following treatment with MK-8776, while there was far less decrease in RPA phosphorylation; some decrease in RPA phosphorylation was expected as resection by Mre11 will also occur downstream of the Mus81-induced DSB.

As the damage induced by MK-8776 was found to occur in replicating cells, we quantified EdU incorporation following treatment with mirin to ensure that the reason for the reduced damage was not slowed DNA replication. We found no difference in the amount of EdU incorporation in untreated cells or those treated with 1 µM MK-8776, 100 µM mirin or 1 µM MK-8776+100 µM mirin for 6 h (data not shown).

Chk1 has been shown to be involved in the regulation of replication origin firing by inhibiting CDK2 activity [7], [10]. In order to determine whether CDK2 activity is involved in the production of DNA damage following Chk1 inhibition, we tested the effects of the CDK2 inhibitor roscovitine on the production of ssDNA and γH2AX in U2OS cells. Roscovitine inhibited the phosphorylation of RPA and γH2AX and prevented the appearance of RPA foci and γH2AX by immunofluorescence (Fig. 3B,C). This is consistent with previous data that roscovitine reduces levels of ssDNA and DSB induced by the Chk1 inhibitor UCN-01 [7].

### Mre11 is required for DNA damage following Chk1 inhibition

**Figure 4:**
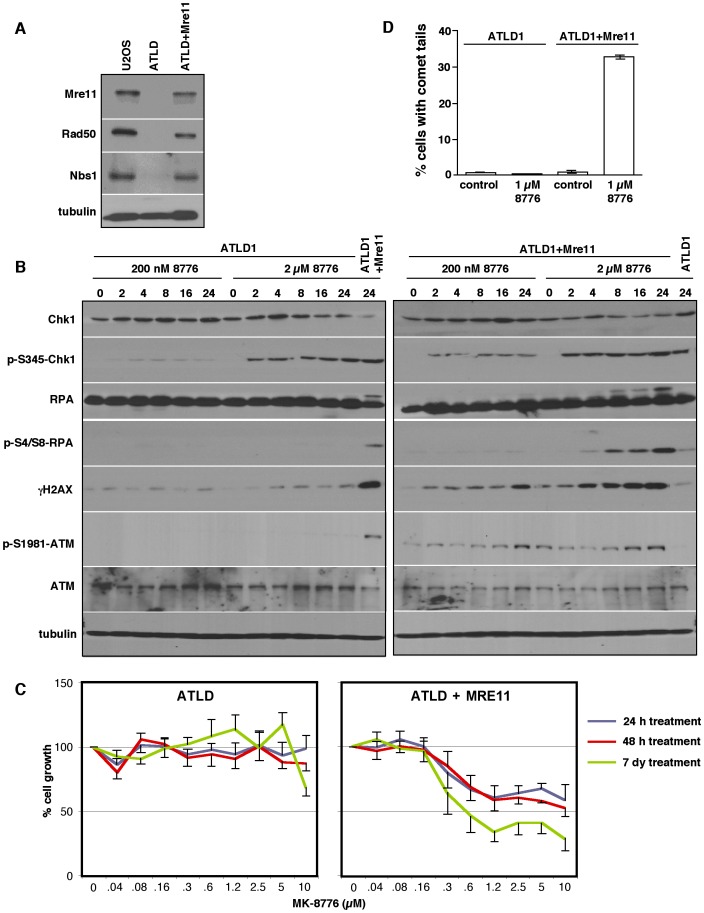
Mre11-deficient ATLD1 cells are resistant to MK-8776. A. Whole cell lysates from U2OS, ATLD1 and ATLD1+Mre11 cells were analyzed by western blot for proteins of the MRN complex. B. ATLD1 and ATLD1+Mre11 cells were incubated with 200 nM or 2 µM MK-8776 for 0–24 h, harvested and analyzed by western blotting. The samples incubated for 24 h at 2 µM were also run beside the samples from the opposite cell line to compare signal intensities. C. Cells were incubated with MK-8776 for 24 h or 48 h then allowed to recover in drug-free media, or incubated continuously for 7 days with MK-8776. Total DNA content per well was then assessed as a measure of cell growth. Error bars (shown only in one direction for clarity) represent the standard error of 3 independent experiments. D. ATLD1 and ATLD1+Mre11 cells were incubated for 24 h with and without 1 µM MK-8776. The neutral comet assay was performed and histograms represent cells with DSB. Error bars represent standard error of 2 independent experiments.

**Figure 5:**
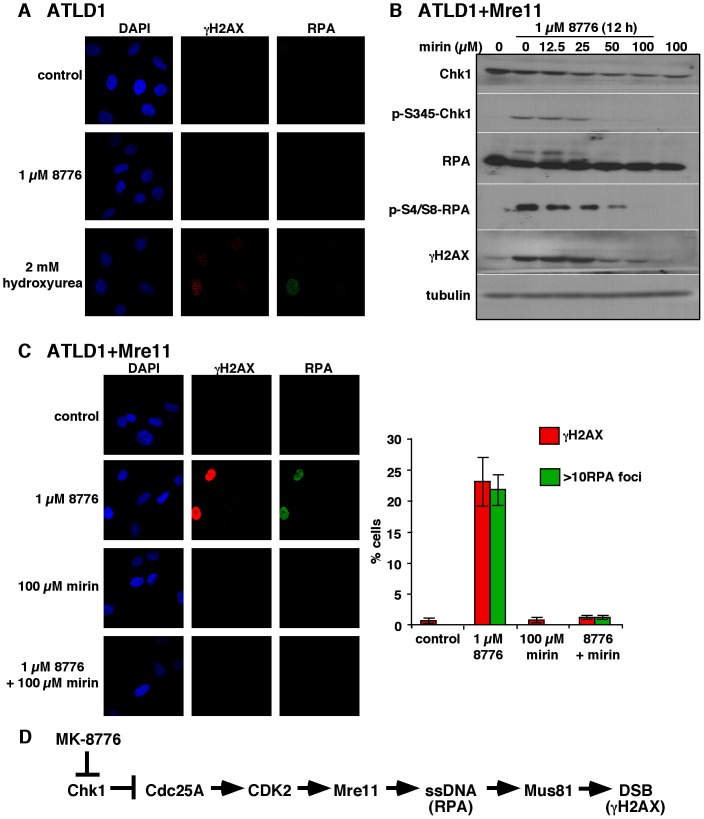
ATLD1 cells do not form RPA or γH2AX foci in response to MK-8776. A. Cells grown on coverslips were incubated with 1 µM MK-8776 for 6 h, or 2 mM hydroxyurea for 24 h, stained with antibodies against γH2AX and RPA and analyzed by confocal microscopy. B. ATLD1+Mre11 cells were incubated with 1 µM MK-8776 for 12 h concurrent with 0–100 µM mirin. Cells were harvested and analyzed by western blotting. C. ATLD1+Mre11 cells grown on coverslips were incubated for 6 h with 1 µM MK-8776, 100 µM mirin or both and analyzed by confocal microscopy for γH2AX and RPA foci. 100 cells were scored for each condition and are presented as histograms on the right. D. Proposed pathway for the induction of damage upon inhibition of Chk1.

Cells from patients with Ataxia-telangiectasia-like disorder (ATLD) express a hypomorphic truncated Mre11 mutation [22]. These cells are hypersensitive to ionizing radiation (IR), fail to suppress DNA synthesis and have a reduced ATM response to irradiation [12]. If Mre11 is required for toxicity induced by Chk1 inhibition, we hypothesized that these cells, lacking functional Mre11, would be inherently resistant to MK-8776. The Mre11 mutation in ATLD1 cells also leads to dramatically reduced expression of Rad50 and Nbs1 [23] which was confirmed by western blotting for these three proteins (Fig. 4A). In these cells, MK-8776 induced negligible levels of γH2AX, phospho-RPA and phospho-ATM in comparison with that seen in U2OS cells (Fig. 4B) and no γH2AX or RPA foci were detectable by immunofluorescence (Fig. 5A, Row 2). These cells are extremely resistant to MK-8776 and continue to grow even during 7 days continuous exposure to the drug (Fig. 4C). Hydroxyurea is an inhibitor of deoxyribonucleotide production via the inhibition of ribonucleotide reductase. H2AX phosphorylation and RPA accumulation on the DNA in response to hydroxyurea is not dependent on Mre11 [24], [25]. For this reason, 2 mM hydroxyurea was used as a control to show that ATLD1 cells retain the ability to induce γH2AX and RPA foci (Fig. 5A, Row 3).

To confirm that the MK-8776 resistance exhibited by ATLD1 cells was due to a lack of Mre11, we used cells complemented with wild type Mre11 cDNA to ectopically express a functional Mre11 protein, which also results in increased Rad50 and Nbs1 (Fig. 4A). These cells regained the ability to phosphorylate RPA, ATM and γH2AX in response to MK-8776 (Fig. 4B). As ATLD1 cells do not activate ATM in response to MK-8776, this could explain the lack of γH2AX in these cells. However, the neutral comet assay demonstrated that MK-8776 did not induce DSB in these cells demonstrating that the lack of γH2AX was not just due to lack of ATM signaling but to a lack of DSB (Fig. 4D). In contrast, approximately 30% of the ATLD1+Mre11 cells exhibited comet tails indicating DSB following treatment with MK-8776.

RPA foci and pan-nuclear γH2AX staining were seen in the ATLD1+Mre11 cells at similar levels to those in the U2OS cells (Fig. 5C). Once again, mirin almost completely abrogated both γH2AX pan-nuclear staining and RPA foci formation (Fig. 5B and C). Cytotoxicity assays showed that a 24 h incubation of ATLD+Mre11 cells with MK-8776 suppressed growth at a similar concentration as U2OS cells, but this only achieved about 50% growth inhibition (Fig. 4C). This is most likely due to the much slower doubling time of these cells (64 h) such that far fewer cells are in S phase during the treatment period. Continuous incubation caused greater growth suppression but did not decrease the concentration of drug required. The wild type ATLD cells have a doubling time of 48 h but appear completely resistant to continuous incubation with MK-8776 demonstrating that their resistance is not due to a slower growth rate. Collectively, these results demonstrate that Mre11 is necessary for the DNA damage induced following Chk1 inhibition and that the ectopic expression of Mre11 can induce damage in resistant, Mre11-deficient cells.

## Discussion

We have shown that the DNA damage induced by the Chk1 inhibitor MK-8776 is dependent on the action of the Mre11 nuclease. In particular we have demonstrated that inhibition of Mre11 activity prevents formation of ssDNA and DSB induced by MK-8776 and UCN-01. These data and the fact that an Mre11-deficient cell line is resistant to Chk1 inhibition, suggests that Mre11 is involved in the production of DNA damage following treatment with Chk1 inhibitors.

Following treatment with MK-8776 there is rapid phosphorylation of Chk1 at the serine-345 site. This is consistent with the report that Chk1 induces feed-back dephosphorylation of ATR by protein-phosphatase 2A; when Chk1 is inhibited, ATR becomes active and phosphorylates Chk1 [16]. This Chk1 phosphorylation was also seen in the ATLD cells in response to MK-8776, indicating that the resistance to MK-8776 in this cell line is not attributable to a failure to inhibit Chk1.

Our study shows that Chk1 inhibition induces rapid phosphorylation of RPA and H2AX in U2OS cells, indicative of the appearance of regions of ssDNA and DSB respectively. We also found that MK-8776 treatment induces RPA foci and γH2AX pan-nuclear staining in about 25% of the cells. Pan-nuclear γH2AX staining, as opposed to foci has been previously documented in response to Chk1 inhibition and has been shown to be associated with DSB following Chk1 inhibition [7]. It has been suggested that this widespread phosphorylation of H2AX is due to the inappropriate firing of replication origins, leading to fork collapse at numerous points distributed throughout the genome [26]. The comet assay was performed to further confirm that DSB were responsible for the phosphorylation of H2AX. Flow cytometry revealed that the cells expressing the phosphorylated form of H2AX were in S phase of the cell cycle. This is consistent with previous reports that Chk1 is required in normal S phase to protect against DNA breakage [7].

In response to DSB induced by ionizing radiation (IR), the MRN complex acts as a DNA damage sensor and is responsible for recruiting ATM to the sites of DNA damage [19]. The MRN complex promotes the initial processing of DSB to ssDNA by the action of the Mre11 nuclease in concert with its partner CtIP [27]. We therefore hypothesized that Mre11 could be involved in the production of ssDNA following Chk1 inhibition as it has been shown to promote the processing of DSB to ssDNA following IR [13]. Inhibition of Mre11 did indeed reduce the amount of ssDNA but importantly Mre11 inhibition also led to a decrease in γH2AX and a decrease in DSB. It is of note that while levels of phospho-RPA and γH2AX were dramatically decreased by the presence of mirin, phospho-Chk1 levels were not altered, indicating that the ability of MK-8776 to inhibit Chk1 was not affected.

The DNA endonuclease Mus81 is required for DSB following Chk1 inhibition [11]. Mus81 is a substrate-specific endonuclease which cleaves structures mimicking Holliday junctions [28] but has been shown to cleave model replication forks and other “nicked” substrates 75-fold more efficiently than stationary Holliday junctions [29]. Our data demonstrate that ssDNA occurs upstream of DSB following Chk1 inhibition and we therefore hypothesize that Mre11 may act to provide the substrate for Mus81 cleavage.

Chk1 controls the cell cycle by targeting the protein phosphatase Cdc25A for degradation [30]–[32], thereby preventing Cdc25A from dephosphorylating and activating CDK2 [33]. Following Chk1 inhibition, Cdc25A accumulates leading to increased active CDK2. The means by which Mre11 is activated following Chk1 inhibition remains unclear. CtIP promotes DSB end resection and has been shown to interact with the MRN complex [34]. CtIP is phosphorylated by CDK2 [35] and DSB resection by MRN/CtIP has recently been shown to be CDK2 dependent in Xenopus eggs [36]. CDK2 has recently been reported to bind directly to Mre11 and this interaction is required for CtIP phosphorylation [37]. Following initial resection by MRN/CtIP, efficient long range strand resection is taken over by the DNA nucleases Exo1 and Dna2. The activation of these two nucleases is also mediated by phosphorylation by CDK2 [38]. The ssDNA produced by these nucleases is then coated with RPA which recruits ATR and subsequently activates Chk1 [13], [39]. These findings taken together with the fact that Chk1-induced DNA damage is prevented by the addition of the CDK inhibitor roscovitine, and by depletion of Cdc25A [5] led us to propose a pathway for the induction of DNA damage following Chk1 inhibition (Fig. 5D). In this pathway, the aberrant activation of CDK2 following Chk1 inhibition leads to activation of Mre11.

There are still many unknown steps in this pathway; in particular why CDK2 does not activate Mre11 in normal S phase. One possibility is that Mre11 activity is suppressed in normal S phase by other factors; for example the Ku and RPA proteins have both been shown to regulate the nuclease activity of the MRN complex [40]. The ability of BRCA2 to stabilize Rad51 filaments has also been shown to protect stalled replication forks from degradation by Mre11 [41]. As Chk1 is required for the interaction of BRCA2 and Rad51 [42], it is possible that in the absence of Chk1 activity, BRCA2 is unable to protect stalled forks from Mre11 degradation. Another possible explanation is that, as Chk1 restrains CDK activity in normal DNA replication [43], the high levels of CDK2 which result from the inhibition of Chk1 in normal S phase could result in over-activation of Mre11. Further work is required to elucidate the finer points of this pathway but it will be of importance to determine whether Mre11 function could act as a determinant of cellular sensitivity to Chk1 inhibitors.

In our recent analysis, we observed a large range of sensitivity to MK-8776 following a 24 h incubation [15]. Interestingly some cell lines resembled the ATLD1 cells in that they continued to grow when incubated with MK-8776. However, these cells are not deficient in Mre11 [44], suggesting there must be alternate mechanisms by which cells can avoid growth inhibition when Chk1 is inhibited. We anticipate that the other steps in the pathway described in Figure 5D can also vary between cell lines, and thereby avoid activation of Mre11. It is also worth recognizing that many previous studies have used U2OS cells as if they are representative of the majority of cell lines, yet our results suggest that in comparison to many other cell lines, U2OS are very sensitive to short-term pharmacologic inhibition of Chk1 [15]. If normal cells are relatively resistant to MK-8776 as has been suggested [45], our hope is that some tumors will be highly responsive and thereby provide a therapeutic window for successful administration of Chk1 inhibitors to patients.

## Materials and Methods

### Human cell lines

U2OS osteosarcoma cells (ATCC, Manassas, VA) were grown in DMEM/F12 and supplemented with 10% fetal bovine serum (FBS). ATLD1 and ATLD+Mre11 cells were a kind gift from Dr. Matthew D. Weitzman at the Salk Institute (San Diego, CA) and were grown in DMEM with 20% FBS. All media also contained 1% antibiotic/antimitotic solution (Gibco, Carlsbad, CA). Cells were grown at 37°C with 5% CO_2_.

### Reagents

Mirin was synthesized in our laboratory as previously described [46], dissolved in DMSO and stored at −20°C. MK-8776 was provided by Merck (Kenilworth, NJ) [45]. Hydroxyurea was obtained from Sigma Chemical Co. (St. Louis, MO).

### Immunoblotting

Cells were rinsed in phosphate buffered saline and lysed by the addition of Laemmli sample buffer. Samples were immediately boiled for 5 min and stored at −20°C. Proteins were separated by polyacrylamide gel electrophoresis and transferred to polyvinylidine difluoride membranes. Membranes were blocked in 5% non-fat milk in Tris buffered saline (TBS), 0.1% Tween 20 and probed with antibody overnight at 4°C in 5% bovine serum albumin/TBS/Tween for phosphospecific antibodies or 5% milk/TBS/Tween for all other antibodies. The primary antibodies used were as follows: phosphoserine-345-Chk1, γH2AX, Nbs1 (Cell Signaling, Danvers, MA); phosphoserines-4/8-RPA32 (Bethyl Laboratories, Montgomery, TX); Chk1 (Santa Cruz Biotechnology, Santa Cruz, CA); RPA32 (Neomarkers, Fremont, CA), Mre11 (Calbiochem, San Diego, CA); Rad50 (Novus Biologicals, Littleton, CO); ATM (AbCam, Cambridge, MA); phoshoserine-1981-ATM (Epitomics, Bulingame, CA). Subsequently, membranes were washed in TBS, 0.1% Tween 20 and incubated with secondary antibody conjugated to horseradish peroxidase (Bio-Rad, Hercules, CA). Proteins were visualized by enhanced chemiluminescence (Amersham, Piscataway, NJ).

### Immunofluorescence

Cells were cultured on glass coverslips, fixed with 3% paraformaldehyde (10 min at room temperature), permeabilized in TBS, 0.5% Triton-X-100 (5 min at room temperature), blocked for 30 min with 10% bovine serum albumin (BSA) and stained with antibodies against RPA32 (Neomarkers) and γH2AX (Cell Signaling) followed by DNA staining with DAPI (5 min, 1 µg/ml in PBS). Confocal images were acquired using a Zeiss LSM 510 microscope. To measure induction of ssDNA, cells were labeled with BrdU (3.3 µM, 24 h), fixed, permeabilized and blocked as described above then stained with anti-BrdU (Becton-Dickinson, Franklin Lakes NJ); the usual DNA denaturation step was omitted so that the antibody only detected endogenous ssDNA.

### Neutral comet assay

Cells were harvested by scraping and suspended at 2×10^5^/ml. The neutral comet assay (Trevigen, Gaithersburg, MD) was performed according to the manufacturer's instructions and 100 cells for each condition were analyzed using the Tri-Tek CometScore™ freeware v1.5. Average tail moment is the usual measure for the comet assay. However, as treatment with MK-8776 only induced damage in S phase cells, results are expressed as the percent of cells with an increase in tail moment over untreated control rather than the average tail moment of the entire population. This was assessed by selecting a tail moment in the untreated control, and then scoring the number of cells in the treatment groups with a higher tail moment. Examples of comet tails are shown in Fig. 2E.

### Transfections and siRNAs

Transfections were performed using RNAi-MAX (Invitrogen, Grand Island, NY) according to the manufacturer's instructions and experiments were performed 48 h afterwards. siRNA sequences: Control 5′-CUGGGUCACUGGUGUUUGA-tt-3′, Mus81 #1 5′ -UGACCUCUCCAAACCCUCU-tt-3′, Mus81 #2 3′-GGGAGCACCUGAAUCCUAA-tt-3′.

### Cytotoxicity assays

Cells were seeded in 96 well plates at a density of 1000 cells/well and left to adhere overnight. Cells were incubated with MK-8776 for the time specified then washed once with PBS, and allowed to recover in media for 7 days. The cells were washed in PBS, lysed for one hour in sodium chloride-sodium citrate buffer with 0.02% SDS at 37°C, then stained with 1 µg/ml Hoescht dye [47]. DNA content was measured using a fluorescence plate reader.
